# Natural and Induced Tolerance to Hymenoptera Venom: A Single Mechanism?

**DOI:** 10.3390/toxins14070426

**Published:** 2022-06-22

**Authors:** Ana Navas, Berta Ruiz-Leon, Pilar Serrano, Manuel Martí, M Luisa Espinazo, Nadine Blanco, Juan Molina, Corona Alonso, Aurora Jurado, Carmen Moreno-Aguilar

**Affiliations:** 1 UGC Inmunología-Alergología, Reina Sofia University Hospital, University of Cordoba, 14004 Cordoba, Spain; anam.navas.ext@juntadeandalucia.es (A.N.); mb.ruiz.sspa@juntadeandalucia.es (B.R.-L.); mpilar.serrano.sspa@juntadeandalucia.es (P.S.); nadine.blanco.sspa@juntadeandalucia.es (N.B.); jeduar.molina.sspa@juntadeandalucia.es (J.M.); mariac.alonso.sspa@juntadeandalucia.es (C.A.); carmen.moreno.sspa@juntadeandalucia.es (C.M.-A.); 2 GC01 Inmunología y Alergología Maimonides Biomedical Research Institute of Cordoba (IMIBIC), Reina Sofia University Hospital, University of Cordoba, ARADyAL INS Carlos III, 14004 Cordoba, Spain; marialuisa.espinazo@imibic.org; 3 Pharmacology Department, Institute of Molecular Pathology Biomarkers, University of Extremadura (UEx), ARADyAL INS Carlos III, 10071 Cáceres, Spain; mmartia@unex.es

**Keywords:** bee venom immunotherapy, tolerance, Helios protein, kynurenine, anaphylaxis

## Abstract

Inducing tolerance in Hymenoptera-allergic patients, bee venom immunotherapy (BVIT) is a widely accepted method to treat severe allergy to bee stings. In order to increase the existing knowledge on the underlying immunological mechanisms and look for possible biomarkers predictive of efficacy, a group of 20 bee-venom-allergic patients (AG) were thoroughly examined during their first year of BVIT. In addition, the results of treated patients with those of an untreated group of 20 tolerant beekeepers (TG) who had previously shown a firm suppressor-regulatory profile were compared. Tolerance in AG patients was invariably associated with a significant regulatory response characterised by the expansion of Helios^−^ subpopulation and increased IL-10, specific IgG4 (sIgG4), and kynurenine levels. Although specific IgE (sIgE) levels increased transiently, surprisingly, the T helper type 2 (Th2) population and IL-4 levels rose significantly after one year of immunotherapy. Thus, the picture of two parallel phenomena emerges: a tolerogenic response and an allergenic one. Comparing these results with those obtained from the TG, different immunological mechanisms appear to govern natural and acquired tolerance to immunotherapy. Of particular interest, the kynurenine levels and T regulatory (Treg) Helios^−^ population could be proposed as new biomarkers of response to BVIT.

## 1. Introduction

Allergy to Hymenoptera venom (bees and wasps) manifests clinically as episodes of anaphylaxis after stings, which can be life-threatening for sensitive subjects. In the case of bee venom, the disease is particularly serious. Bee venom immunotherapy (BVIT) is the only known tool capable of reversing the risk in these patients [1]. Some highly exposed beekeepers with prolonged exposure develop a natural tolerance to stings [2]. In previous work, we compared the immunological status of a group of these tolerant subjects with a group of untreated allergic subjects who had developed systemic reactions to stings. Therefore, we found that the beekeepers developed a weak allergic response, marked by the presence of specific IgE (sIgE) to venom and its allergenic components and a moderate response in the basophil activation test (BAT), at the same time as a robust regulatory-suppressive response, marked by the production of specific IgG4 (sIgG4) to venom and its components, the production of IL-10, and the expansion of the CTLA4^+^ and Helios^−^ regulatory populations. At the other extreme, allergic patients showed intense sIgE production together with a potent BAT response and reduced sIgG4 levels in addition to CTLA4^+^ and Helios^−^ expression [3]. It has been previously described that the immune response to Hymenoptera venom immunotherapy is characterized by the development of the sIgG4 response to the detriment of the sIgE response [4] and an important role for IL-10 as a crucial element of the regulatory response [5]. In this context, and based on the differences found between allergic patients and tolerant beekeepers, this study aimed to determine the changes induced by BVIT over a year concerning the acquired protection against stings and to compare them with the mechanisms of tolerance acquired naturally by a group of highly exposed beekeepers. We looked with particular interest for those factors that, in the future, could be explored in routine clinical practice as biomarkers of response. This study is part of a larger project that analyses sting tolerance before immunotherapy, during immunotherapy at different times, and after its discontinuation.

## 2. Results

### 2.1. Biodemographic and Clinical

A total of 20 patients partially overlapped with those in reference [3], comprised the allergic group AG, 15 (75%) of whom were male. Their age at recruitment was 40.4 ± 13.95 years. Twelve (60%) performed full or part-time beekeeping work, nine of them (45%) for ten years or more. A history of atopy was demonstrated in nine patients (45%). Thirteen patients (65%) presented grade 3 anaphylaxis after a bee sting, and the remaining seven (35%) had grade 2 anaphylaxis. Only two patients had a REMA-Score >2, confirming indolent systemic mastocytosis. When serial intradermal reaction with bee venom was performed, in three cases (15%), it was positive at 0.001 µg/mL: in 11 (55%) at 0.01 µg/mL and in 4 (20%) at 0.1 µg/mL. Two patients had a negative skin response to the maximum concentration tested. The sIgE-AmV in the AG was 7.47 (0.22–238) IU/mL, and the sIgG4-AmV median value 0.18 (0–4.48) µg/mL. All patients received BVIT, eight (40%) with the conventional maintenance dose of 100 µg, one (5%) with 150 µg after having presented an episode of Kounis syndrome with this dose, five (25%) with 200 µg, and six (30%) with 300 µg. Two patients (10%) suffered a single systemic reaction during up-dosing. One of them presented a generalized cutaneous episode, and the other (meeting criteria for mastocytosis), an episode of Kounis syndrome. There were no reactions in the maintenance phase. At the end of the first year of BVIT (T3), sting challenge test (SCT) was performed in 19 patients, all with negative results. One patient did not give her authorization for SCT although she authorized the rest of the study interventions. The individual AG data are shown in Table 1. The 20 individuals in the TG were studied at a single time, and their results have been partially published previously [3]. Their individual data are shown in Table 2.

### 2.2. Efector Cells

**Figure 1:**
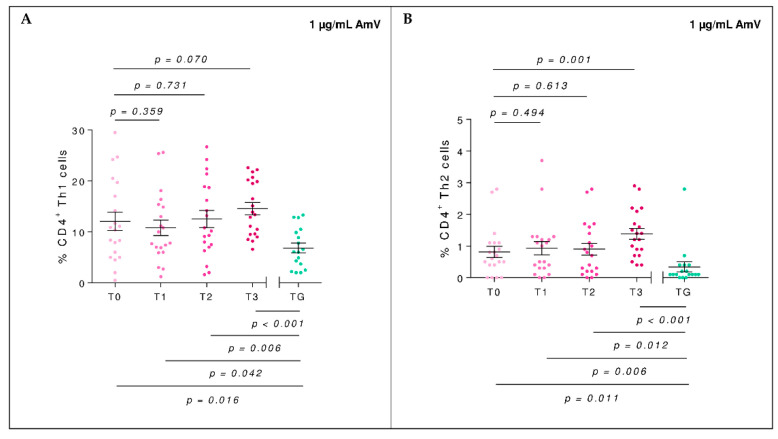
Percentage of CD4^+^Th1 (**A**) and Th2 (**B**) subsets of the allergic group (AG, pink) throughout the BVIT course and tolerant group (TG, green) when priming the culture with 1 µg/mL of AmV. The mean and standard error of the mean bars is displayed. At the top of the plot, mixed-effect model *p*-values of time are shown, establishing T0 as the baseline level, whereas at the bottom, unpaired *t*-tests (for equal or unequal variances) or Mann–Whitney U tests to compare the mean in each time respect to TG are considered.

T helper type 1 (Th1) population did not differ significantly between baseline time (T0) and first-year BVIT-sting challenge test (T3) in AG (*p* = 0.070, t_me_ = 1.85 in mixed-effect model). However, Th2 was significantly increased at T3 (*p* = 0.001, t_me_ = 3.41). When comparing the values of these effector populations between AG and TG, the differences found at T0 (Th1: *p* = 0.016, t = −2.55 in unpaired *t*-test and Th2: *p* = 0.011, W = 87 in Mann–Whitney U test with the rank sum statistic) persisted at T3 (Th1 and Th2: *p* < 0.001, t = −4.88 and W = 25.5, respectively) (Figure 1).

### 2.3. T-Regulatory Populations

**Figure 2:**
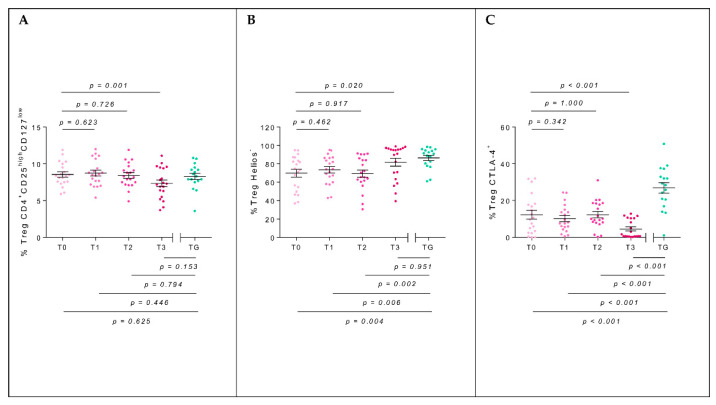
Percentage of peripheral blood CD4^+^CD25^high^CD127^low^ (**A**), Helios^−^ (**B**), and CTLA-4^+^ (**C**) Treg cells of the allergic group (AG, pink) throughout the BVIT course and tolerant group (TG, green). The mean and standard error of the mean bars are displayed. At the top of the plot, mixed-effect model *p*-values of time are shown, establishing T0 as the baseline level, whereas at the bottom, unpaired *t*-tests (for equal or unequal variances) or Mann–Whitney U tests to compare the mean in each time respect to TG are considered.

The T regulatory (Treg) Helios^−^ population in AG showed a significant increase between T0 and T3 (*p* = 0.020, t_me_ = 2.40). Moreover, the difference existing at T0 with TG (*p* = 0.004, W = 265.5) disappeared at T3 (*p* = 0.951, W = 168). The cells CD4^+^CD25^high^CD127^low^ and CTLA4^+^ significantly decreased between T0 and T3 (*p* = 0.001, t_me_ = −3.40 and *p* < 0.001, t_me_ = −3.50, respectively). Concerning the comparison between AG and TG, no significant differences were observed at T3 for CD4^+^CD25^high^CD127^low^ (*p* = 0.153, t = 1.46), but it did for CTLA4^+^ (*p* < 0.001, W = 330) (Figure 2A–C).

### 2.4. Basophil Activation Test

**Figure 3:**
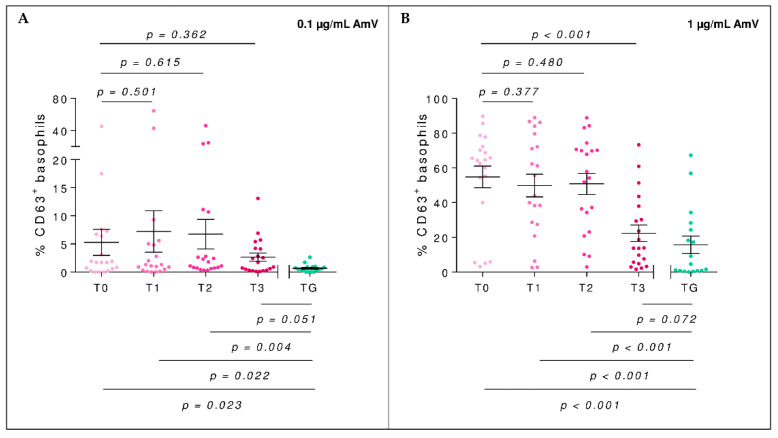
Percentage of activated basophils (%CD63^+^) of the allergic group (AG, pink) throughout the BVIT course and tolerant group (TG, green) when using 0.1 µg/mL (**A**) and 1 µg/mL (**B**) of AMv as a stimulus. The mean and standard error of the mean bars is displayed. At the top of the plot, mixed-effect model *p*-values of time are shown, establishing T0 as the baseline level, whereas at the bottom, unpaired *t*-tests (for equal or unequal variances) or Mann–Whitney U tests to compare the mean in each time respect to TG are considered.

The percentage of basophil activation in AG was significantly reduced between T0 and T3 (*p* < 0.001, t_me_ = −5.87). Although at T0, there was a significant difference with TG (*p* < 0.001, W = 45), at T3, no such difference was observed (*p* = 0.072, W = 111) (Figure 3A,B).

### 2.5. Interleukins

**Figure 4:**
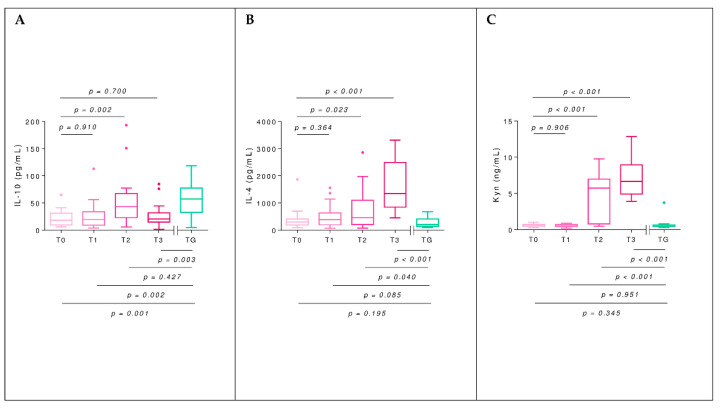
Box-plots for the IL-10 (**A**) and IL-4 (**B**) levels (pg/mL) quantified in culture supernatant when priming the culture with 1 µg/mL of AmV and plasma kynurenine level (Kyn; ng/mL) (**C**) of the allergic group (AG, pink) throughout the BVIT course and tolerant group (TG, green). At the top of the plot, mixed-effect model *p*-values of time are shown, establishing T0 as the baseline level, whereas at the bottom, unpaired *t*-tests (for equal or unequal variances) or Mann–Whitney U tests to compare the mean in each time respect to TG are considered.

IL-10 values in AG experienced a significant increase when maintenance dose was reached (T2) (*p* = 0.002, t_me_ = 3.23) that did not persist at T3 (*p* = 0.700, t_me_ = 0.39). Regarding the comparison between AG and TG, at T0, both groups showed a significant difference (*p* = 0.001, W = 227), approached similarity at T2 (*p* = 0.427, W = 176), and were again significantly different at T3 (*p* = 0.003, W = 252). IL-4 in AG increased progressively, reaching a significant difference concerning the basal time at T2 (*p* = 0.023, t_me_ = 2.35); this difference persisted at T3 (*p* < 0.001, t_me_ = 7.34). The IL-4 comparison between AG and TG did not differ at T0 (*p* = 0.195, W = 100); nevertheless, they were significantly different at T2 (*p* = 0.040, W = 90) and maintained this difference at T3 (*p* < 0.001, W = 6) (Figure 4A,B).

### 2.6. Kynurenine

The kynurenine values in AG were significantly elevated at T2 concerning T0, with this difference maintained at T3 (*p* < 0.001 in both cases, t_me_ = 7.34 and 11.21, respectively). The comparison between AG and TG showed statistical similarity at T0 (*p* = 0.345, W = 139), reached a significant difference at T2, and maintained it at T3 (*p* < 0.001 in both cases, W = 34 and 0, respectively) (Figure 4C).

### 2.7. Specific Immunoglobulin E

sIgE-AmV in AG was elevated between T0 and T2 and decreased at T3 without reaching statistical significance. At T1, T2, and T3 AG maintained significantly higher values than TG (*p* < 0.001 in all cases, W = 71, 28, and 77, respectively). An elevation of sIgE was observed at T2 to all the allergenic components studied, being non-significant for rApi m1 (*p* = 0.096, t_me_ = 1.69) and significant for the remaining ones: rApi m2 (*p* = 0.024, t_me_ = 2.31), rApi m3 (*p* = 0.007, t_me_ = 2.77), Api m 4 (*p* = 0.024, t_me_ = 2.31), rApi m5 (*p* = 0.005, t_me_ = 2.89), and rApi m 10 (*p* = 0.020, t_me_ = 2.39). The decrease in sIgE at T3 was also widespread although without statistical significance: rApi m 1 (*p* = 0.394, t_me_ = −0.86), rApi m 2 (*p* = 0.905, t_me_ = 0.12), rApi m 3 (*p* = 0.781, t_me_ = −0.28), Api m 4 (*p* = 0.256, t_me_ = −1.15), rApi m 5 (*p* = 0.258, t_me_ = −1.14), and rApi m 10 (*p* = 0.433, t_me_ = −0.79). When sIgE values of AG subjects at T3 were compared with those at TG, they maintained significantly lower values of sIgE-rApi m 1 (*p* < 0.001,W = 71), rApi m 2 (*p* = 0.036, W = 277.5), rApi m 3 (*p* = 0.032, W = 126), rApi m 5 (*p* = 0.023, W = 116.5), and rApi m 10 (*p* = 0.029, W = 119.5) and were unchanged for Api m 4 (*p* = 0.817, W = 208.5) (Figure 6A–F).

### 2.8. Specific Immunoglobulin G4 

**Figure 5:**
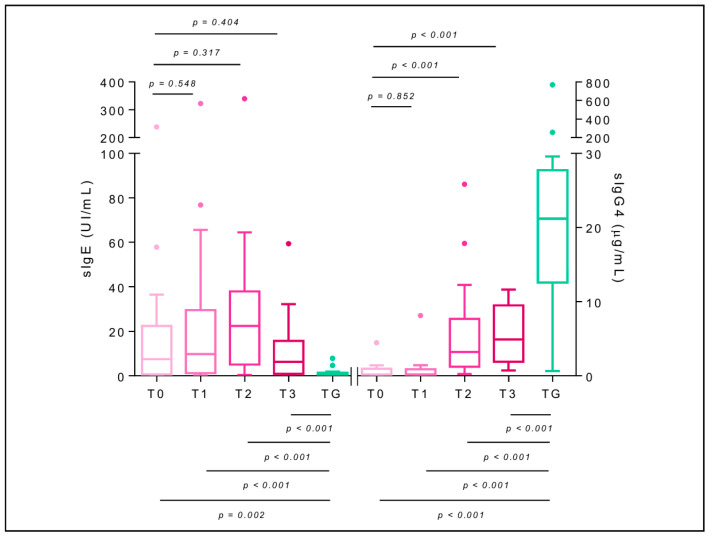
Box-plots for the sIgE level (left *y-axis*) and sIgG4 level (right *y-axis*) to AmV of the allergic group (AG, pink) throughout the BVIT course and tolerant group (TG, green). At the top of the plot, mixed-effect model *p*-values of time are shown, establishing T0 as the baseline level, whereas at the bottom, unpaired *t*-tests (for equal or unequal variances) or Mann–Whitney U tests to compare the mean in each time respect to TG are considered.

**Figure 6:**
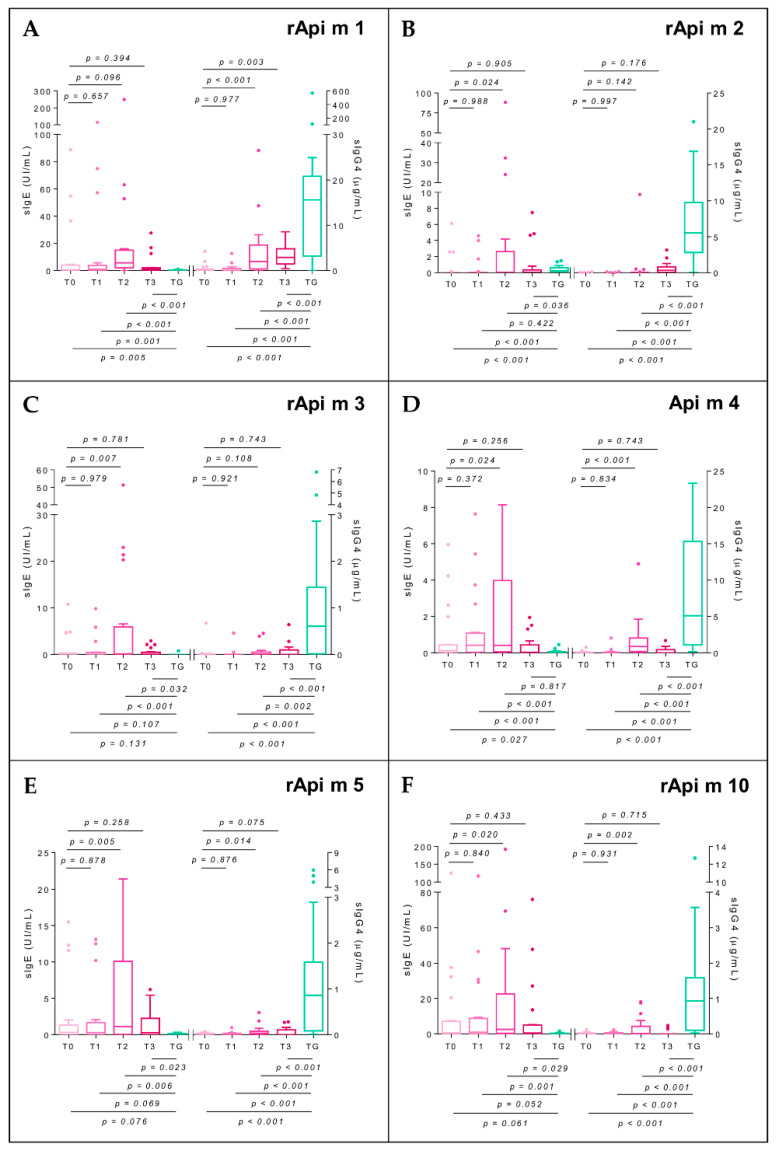
Box-plots for the serum-specific IgE level (sIgE; left *y-axis*) and specific IgG4 level (sIgG4; right *y-axis*) to *Apis mellifera* venom components (rApi m 1, rApi m 2, rApi m 3, Api m 4, rApi m 5, and rApi m 10; (**A**–**F**) panels) of the allergic group (AG, pink) throughout the BVIT course and tolerant group (TG, green). At the top of the plot, mixed-effect model *p*-values of time are shown, establishing T0 as the baseline level, whereas at the bottom, unpaired *t*-tests (for equal or unequal variances) or Mann–Whitney U tests to compare the mean in each time with respect to TG are considered. Table S1 shows the results in detail.

sIgG4-AmV in AG shows a significant elevation at T2 (*p* < 0.001, t_me_ = 5.04) that persists at T3, but the TG group shows significantly higher values at all times of the study (*p* < 0.001, W = 392, 390, and 345, respectively) (Figure 5). Regarding sIgG4 values to allergenic components, an increasing trend is observed at T2 with respect to T0: rApi m 1 (*p* < 0.001, t_me_ = 3.78), rApi m 2 (*p* = 0.142, t_me_ = 1.49), rApi m 3 (*p* = 0.108, t_me_ = 1.63), Api m 4 (*p* < 0.001, t_me_ = 3.64), rApi m 5 (*p* = 0.014, t_me_ = 2.52), and rApi m 10 (*p* = 0.002, t_me_ = 3.27), which is maintained at T3: rApi m 1 (*p* = 0.003, t_me_ = 3.08), rApi m 2 (*p* = 0.176, t_me_ = 1.37), rApi m 3 (*p* = 0.743, t_me_ = 0.33), Api m 4 (*p* = 0.743, t_me_ = 0.33), rApi m 5 (*p* = 0.075, t_me_ = 1.81), and rApi m 10 (*p* = 0.715, t_me_ = 0.37). Differences between AG at T3 and TG remained statistically significant for all the allergenic components (*p* < 0.001) (Figure 6A–F).

## 3. Discussion

In order to increase the existing knowledge on the underlying immunological mechanisms and look for possible biomarkers predictive of efficacy, a group of 20 bee-venom-allergic patients (AG) were thoroughly studied during their first year of BVIT. In addition, we compared the results of treated patients with those of an untreated group of tolerant beekeepers (TG) who had previously shown a firm suppressor-regulatory profile [3]. Overall, we found that AG, in response to BVIT, developed a significant regulatory response characterised by the expansion of Helios^−^, IL-10, and serum sIgG4. In addition, serum Kynurenine values also increased significantly with BVIT, aligning with regulatory influences. sIgE, which experienced an initial rise in AG, showed a downward trend after one year of BVIT (T3) without statistical significance. In contrast, basophil activation was steadily reduced throughout the first year of treatment, and in T3, the results were equal to those observed in TG.

Although the CD4^+^CD25^high^CD127^low^ expressing cells that characterise the regulatory population did not show significant changes with BVIT, the Helios^−^ subpopulation did. The Helios receptor is a transcription factor whose expression in thymus-matured Treg cells (tTreg) has been proposed by some authors as a fundamental element to differentiate a self-antigen-tolerance-inducing population from another population with extrathymic maturation (iTreg) and tolerogenic function against external antigens, namely Helios^−^ [3,6,7,8,9,10].Current data point in the direction that BVIT maintained for one year induces tolerance by a mechanism involving the expansion of the Helios^−^ population and that this phenomenon is also present in the natural tolerance developed by TG. This hypothesis has also been proposed to explain acquired tolerance in the peanut-allergenic immunotherapy (AIT) model [11] but could not be confirmed for AIT with Der p 1 [12].

In contrast to the Helios^−^ subpopulation, in the AG, the expression of CTLA-4 in Treg cells was not modified during the build-up phase (T2), but at T3, there was a dramatic reduction in both mean and individual values. This contrasts with the characteristics of Treg cells in TG, expanded at the expense of Helios^−^ population, which also expresses CTLA-4. The declining behaviour of CTLA-4^+^ regulatory cells or soluble CTLA-4 has been previously observed in models of venom immunotherapy (VIT) [13,14] and other AITs [15]; however, other authors have described an opposite effect [16,17,18]; additionally, in a study analysing the influence of AIT and natural seasonal pollen exposure in allergic patients, different behaviour for CTLA-4 expression in Treg cells and Th2 cells has been reported [19].

The observed difference in CTLA-4 expression in TG versus AG suggests different mechanisms for obtaining tolerance to bee venom through immunotherapy or natural exposure to bee stings. Considering that CTLA-4 is an essential molecule for immunological tolerance [20], a phenotypic dysregulation of CTLA-4^+^ iTreg cells in allergic subjects, similar to that described in patients with systemic lupus erythematosus [21], could be considered.

Irrespective of CTLA-4 expression, the iTreg population contains two functionally relevant subpopulations, one being Th3 TGF-β-producing cells and the other Tr1 IL-10-producing cells. AG patients have developed a potent IL-10 response early in the course of treatment (T2), approaching at that time the TG condition, in accordance with what has been published for VIT [22] and grass AIT [23]. In our case, it is possible that the spacing of the antigenic supply during the maintenance phase of BVIT results in a lower IL-10 response than those produced by the effect of short up-dosing with higher dose concentrations. This observed kinetics of IL-10 production and taking into account the kinetics of changes in the Helios^−^ population suggests the involvement of regulatory actors other than the Tr1 population. B cells, dendritic cells, macrophages, monocytes, and natural killer cells have been shown to have the capacity to secrete IL-10 [24], and recently, it has been described that the clinical response to grass AIT is related to IL-10 production by innate lymphoid cells type 2 [25].

One metabolic route that promotes IL-10 secretion is the Indoleamine 2,3-dioxygenase 1 (IDO1) pathway. IDO1 is an enzyme that catalyses tryptophan degradation; this enzymatic function allows the generation of kynurenine by IDO1^+^ cells, contributing to an immunosuppressive microenvironment characterised by impairment of effector T cells and increased regulatory T-cell activity. Similarly, tryptophan degradation leads to anergy in effector T cells [26]. In addition, kynurenine is an aryl-hydrocarbon receptor agonist, promoting the differentiation of effector T cells into regulatory T cells and the induction of IDO in dendritic cells, making them regulatory [26,27]. Additionally, it has been described that kynurenins can cause cell death of T helper type 1 (Th1) cells while favouring T helper type 2 (Th2) cells, shifting the balance between the Th1-Th2 to Th2 ratio [28]. In our study, kynurenine levels of AG did not differ significantly from TG at T0. However, they rose significantly from T2 onwards, being statistically different from TG values, suggesting that the kynurenine pathway could represent a mechanism of activation of BVIT-induced regulation-suppression but not of the tolerance obtained naturally by the multiple stings suffered by TG. The primary inducer of IDO1 is IFN-γ, which may represent a counterbalancing response under inflammatory conditions [26,28]. This fact might explain the differences found between kynurenine production in naturally tolerant conditions and allergic patients treated with BVIT. It should be noted that, unlike other variables in this study, kynurenine is a serum product that can be easily measured in the clinical laboratory at affordable costs, making it a candidate biomarker for future validation. However, a limitation to its predictive value for tolerance to stings is that all patients in the sample were protected after one year of BVIT, as demonstrated by the negativity of the controlled re-sting test; therefore, comparisons with proven therapeutic failure patients could not be made. Moreover, kynurenine can be elevated in many human diseases such as cancer, infections, Alzheimer’s, Parkinson’s, or amyloidosis [26]. Therefore, its role as a biomarker of tolerance must be assessed in the context of these limitations. 

In our study, the percentage of Th2 lymphocytes (CD4^+^/IL-4^+^) after AmV stimulation did not change overall in T2 although 9 of 20 patients showed a Th2 decrease. At T3, on the other hand, there was a significant increase in Th2 in the AG. IL-4 levels measured in stimulated PBMC culture supernatant increased from T1, reaching statistical significance at T2 and maintaining it at T3. Th1 lymphocytes showed an increasing trend without statistical significance at T2 and T3. This finding does not confirm the commonly accepted Th2-Th1 switch described previously [13,29,30] as a result of studies that were conducted on different schedules than ours. On the other hand, an elevation of IL-4 [31] and unchanged Th2 and Th1 populations [32] after AIT with grasses have been described. These seemingly paradoxical behaviours could be due to methodological differences in the use of Th2 subpopulation markers, the conditions of allergen exposure during immunotherapy, or the challenge model employed (patient boost or laboratory priming) or reflect a variety of mechanisms of immune tolerance production by immunotherapy over time. The Th2 to Th1 switch described in certain AIT models could occur via two pathways. The first refers to the plasticity of Th cells after differentiation into effector cells such that even fully differentiated Th1 and Th2 cells could change their transcriptional signature in the first few days of stimulation, while prolonged stimulation could result in a more stabilised phenotype [33]. The second mechanism could be cross-inhibition between different polarised T helper cells; in this model, different immune responses inhibit each other while self-amplifying [33].

Another tolerogenic mechanism of AIT is the isotype switching from sIgE to sIgG4. It has been previously described that VIT produces an initial increase in sIgE levels followed by a decrease over the years, associated with a progressive increase in sIgG4 [34,35]. In our study, there was an early increase of sIgE at the end of up-dosing to all bee venom molecular components, significant for rApi m 1, rApi m 3, Api m 4, rApi m 5, and rApi m 10, with a decrease evident in T3. In parallel, there was a sustained increase in sIgG4 to all molecular components, which was highly significant in the case of rApi m 1. Our results suggest that the sIgE-sIgG4 switch is not the consequence of exchange between both pathways but the result of two parallel pathways: in the first one, IgE-producing plasma cell clones would tend to decrease, while some memory B lymphocytes would continue to produce IgE in a limited way. There would be a potent stimulation of new clones of IgG4-producing B lymphocytes in the second. This interpretation is in line with the mechanism of immunoglobulin gene recombination, whereby the coding DNA region of the IgE constant fragment is located beyond the IgG gamma chain’s coding regions; therefore, a B cell and all its progeny cannot switch to IgG4 after having switched to IgE. The increase in sIgG4 that occurs during AIT results from new B-lymphocyte clones [36]. Thus, during AIT, clones of plasma cells that maintain sIgE production and clones of memory B cells that are in contact with the allergen are increasing sIgE production. At the same time, due to the inherent characteristics of AIT (frequency of administration, amount of antigen, allergen composition, route of administration), new clones of B lymphocytes that produce sIgG4 would be generated.

One mechanism of action of IgG4 for tolerance induction lies in restraining effector cells (mast cells and basophils) through inhibition of the FcγIIb receptor. Our results show superimposable kinetics in time for the growth of sIgG4 levels, the reduction in the percentage of basophil activation, and the need for a higher concentration of venom to stimulate degranulation. As in other previous works on VIT [37,38,39], basophil activation decreased throughout treatment, with the differences being extremely significant at T3 when the differences with TG were diluted, becoming virtually identical. Basophil degranulation was not an early marker, and there was no significant difference in up-dosing. BAT has been shown to be a marker of great value in the follow-up of BVIT although, unfortunately, its complexity of execution, including the need to perform the technique with fresh blood, makes it unrealistic for clinical practice. However, a recent publication suggests that automation of BAT is possible [40].

One of the main limitations of our study is the sample size. The other limitation is the extraordinary efficacy of the treatment, with a 100% response rate, which makes it difficult to assess the specific weight of each of the findings described in the tolerance process.

## 4. Conclusions

On the whole, the evidence from the monitoring data of this study during and at the end of the first year of BVIT clearly identifies different immunological mechanisms governing natural and acquired tolerance.

Moreover, a picture emerges in which two phenomena occur in parallel. Furthermore, what happens at the cellular level mirrors what happens at the humoral level: a tolerogenic response by production of sIgG4, Helios^−^ Treg cells, desensitized basophils, IL-10, and metabolites of the kynurenine pathway and simultaneously a pro-allergenic response by effector memory T cells and sIgE will need to be closely monitored. Among all of them, kynurenine merits particular attention as a suitable clinical biomarker.

The T2 time at the end of up-dosing is a critical point where tolerance and sensitization phenomena accumulate.

Some of the markers studied have proven helpful for monitoring the early response to BVIT, such as IgG4 to rApi m 1. In contrast, others, such as kynurenine, the basophil degranulation test, or the Treg Helios^−^ cells, are useful for the late phase.

## 5. Materials and Methods

### 5.1. Study Design

**Figure 7:**
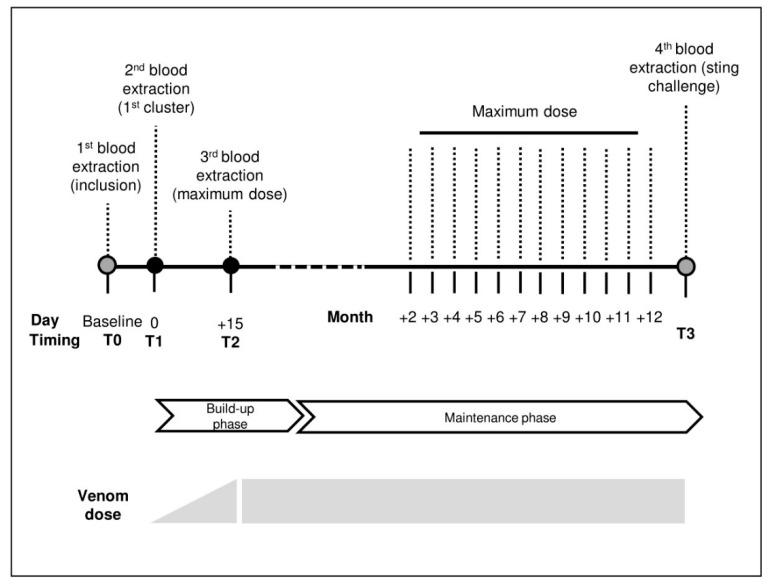
Timeline of the study for the treated allergy group.

In this longitudinal prospective trial, a total of 20 patients (allergic group, AG) older than 18 years were included. All of them suffered at least one episode of anaphylaxis after a bee sting and were transferred for allergy diagnosis and treatment between January and December 2016. Allergy to (AmV) was confirmed by conventional practice (intradermal test positive at least to 0.1 µmg/mL (Pharmalgen *A. mellifera*, ALK, Madrid, Spain) and sIgE ≥0.35 UI/mL to AmV (ImmunoCAP, Thermofisher, Uppsala, Sweden). After diagnosis, they were considered candidates for BVIT according to the protocol of the centre. We include the follow-up of the first year of BVIT. See Figure 7.

Additionally, a total of 20 beekeepers receiving more than 50 stings/year for more than ten years without experiencing extensive local or systemic reactions were included in the study as a tolerant group (TG). They did not receive BVIT.

### 5.2. Demographic and Clinical Features

We collected data on AG individuals: sex, age, professional activity related to stings exposure, atopy (accepted as demonstrated atopic dermatitis, rhinoconjunctivitis, asthma or urticaria, and positive skin prick test or sIgE ≥ 0.35 UI/mL to aerial or food allergens in relationship with clinical symptoms), mastocytosis approach based on REMA score [41], in addition to the severity of sting induced anaphylaxis (grades 1–3) according to the EAACI classification [42].

### 5.3. Biological Samples

Blood samples from AG were taken four times according to the schedule included in Figure 7. Blood samples from TG were taken once out of the beekeeping season.

### 5.4. Immunotherapy

BVIT was indicated according to international guidelines [1] and administered in all cases by trained nurses in controlled conditions under the direct supervision of an allergist, following the usual clinical protocol of the centre, with Pharmalgen^R^ (ALK, Madrid). The build-up phase was based on a cluster schedule to reach the therapeutic dose in 2, 3, or 4 visits (Figure S1). Maintenance therapy consisted of Pharmalgen^R^ 200 μg for patients with a high risk of exposure to bee stings and 100 μg otherwise. However, an increased maintenance dose of 300 μg was administrated to patients who were predominantly sensitized to allergens with a scarce presence in the AmV. The evaluation was conducted during the first year of treatment.

### 5.5. Evaluation of BVIT Safety

Occurrence (yes/no) and the number of systemic reactions (SRs) during induction of immunotherapy were recorded using the EAACI grading [42]. All patients began BVIT without premedication to avoid confounding effects on the occurrence of SRs. Patients who experienced a systemic reaction during the build-up phase of therapy were pre-treated with dexchlorpheniramine (5 mg) and methylprednisolone (1 mg/kg) at each subsequent administration. 

### 5.6. Sting Challenge

An in-hospital SCT with a live honeybee was offered to AG to evaluate treatment efficacy. The test was carried out after one year of BVIT, as described for vespids [43]. Responses were classified using the EAACI system [42]. Patients with a negative result remained under observation for 2 h after the challenge.

### 5.7. Serum sIgE and sIgG4 Level to Apis Mellifera Venom and Its Molecular Components

Serum sIgE and sIgG4 to AmV and all its components (rApi m 1-phospholipase A2, rApi m 2-hyaluronidase, rApi m 3-acid phosphatase, Api m 4-mellitin, rApi m 5-dipeptidil-peptidase, and rApi m 10-icarapin) were measured by fluoroimmunoassay with ImmunoCAP 250 (Thermofisher, Uppsala, Sweden) from samples obtained from AG and TG and preserved at −20 °C until being processed. All the assays were performed according to manufacturer instructions. In order to quantify the sIgE and sIgG4 levels to Api m 4 (Melittin Sequence: H-GIGAVLKVLTTGLPALISWIKRKRQQ-OH from Schafer-N ApS, Denmark), it was coupled to activated CAPs by Thermofisher Scientific [44].

### 5.8. Basophil Activation Test

(BAT) was assessed using 100 µL of heparinized total blood samples from AG and TG and whole AmV (Pharmalgen^R^, ALK, Madrid, Spain) as stimulus at two different concentrations (0.1 µg/mL and 1 µg/mL). Phosphate-buffered saline (PBS) and N-Formyl-Met-Leu-Phe (fMLP; ref. f3506 Sigma-Aldrich, Sal Luis, MO, USA) at a concentration of 2 µM were used as negative and positive controls, respectively. In brief, blood samples were pre-incubated with Basophil Stimulation Buffer (ref. 339664, Becton, Dickinson and Company, San Jose, CA, USA) for 10 min at 37 °C. Then, negative and positive control and AmV were added in separate tubes. All tubes were incubated for 30 min at 37 °C. Basophil degranulation was stopped by transferring samples to an ice bath for 5 min. Cell staining was performed using CD63-FITC/CD123-PE/anti-HLA-DR-PerCP cocktail (ref. 341068, BD FastImmune^TM^, Becton, Dickinson and Company, San Jose, CA, USA). After lysing cells with 2 mL of 1x BD FACS^TM^ lysing solution and washing twice with PBS, stained cells were acquired in a BD FacsCanto II cytometer (Becton Dickinson and Company, San Jose, CA, USA), using BD FacsDiva^TM^ as acquisition and analysis software. At least 500 events CD123^+^ were recorded. The basophil degranulation was measured as the percentage of basophils expressing the surface marker CD63 (%CD63^+^) (Figure S2).

### 5.9. T-Cell Phenotype

Th1, Th2, and Th17 lymphocyte subsets were identified according to their cytokine secretion profile using the human Th1/Th2/Th17 Phenotyping Kit (ref. 560751, BD Pharmingen^TM^, Becton, Dickinson and Company, San Jose, CA, USA). For this purpose, 1:1 PBS-diluted heparinized total blood was stimulated for 5 h at 37 °C and 5% CO_2_ using phorbol 12-myristate 13-acetate (ref. P1585, Sigma-Aldrich) at 50 ng/mL, ionomycin calcium salt (ref. I0634, Sigma-Aldrich) at 1 µg/mL, and AmV (Pharmalgen^R^, ALK, Madrid, Spain) at 1 µg/mL in the presence of BD GolgiStop^TM^ Protein Transport Inhibitor (provided in the kit). Once stimulated, cells were collected, fixed, and permeabilized before the staining, according to the manufacturer’s instructions. The staining was performed using CD4-PerCP-Cy5.5/IL-17A-PE/INF-GMA-FITC/IL-4-APC cocktail included in the kit (Figure S3).

T-regulatory cell phenotype was performed using 100 µL of whole blood, using the Transcription Factor Buffer Set kit (ref. 562574, BD Pharmingen^TM^, Becton, Dickinson and Company, San Jose, CA, USA) and 50 µL of Brilliant Stain Buffer (ref. 566349, BD Horizon, Becton, Dickinson and Company, San Jose, CA, USA), according to the manufacturer protocol, with the following combination of monoclonal antibodies (Figure S4): CD3-BB515 (ref. 564465, BD Horizon), CD4-APC-H7 (ref. 560158, BD Pharmingen), CD25-PE-Cy7 (ref. 557741, BD Pharmingen), CD127-AlexaFluor647 (ref. 558598, BD Pharmingen), CD39-BV421 (ref. 563679, BD Horizon), CD45RA-BV510 (ref. 563031, BD Horizon), CTLA-4-PE (ref. 555853, BD Pharmingen), CD8-PerCP-Cy5.5 (ref. 565310, BD Pharmingen), Ki-67-BV421 (ref. 562899, BD Horizon), and Helios-PE (ref. 563801, BD Pharmingen).

Sample acquisition was performed in a BD FacsCanto II cytometer (Becton Dickinson and Company, San Jose, CA, USA), and BD FacsDiva^TM^ was used as acquisition and analysis software. At least 20,000 CD4^+^ lymphocytes were acquired (Figure S4).

### 5.10. IL-4 and IL-10 Cytokines and Kynurenine Quantitation

The production of IL-4 and IL-10 cytokines was assessed from the supernatant of cell culture performed with 1:1 PBS-diluted heparinized total blood samples stimulated for 24 h at 37 °C and 5% CO_2_ using phorbol 12-myristate 13-acetate (ref. P1585, Sigma-Aldrich) at 50 ng/mL, ionomycin calcium salt (ref. I0634, Sigma-Aldrich) at 1 µg/mL, and AmV (Pharmalgen^R^, ALK, Madrid, Spain) at 1 µg/mL. After centrifuging, the supernatant was collected and frozen at −80 °C until being analysed. The measurement of cytokines was performed using the customized Milliplex^®^ Map Human High Sensitivity T-Cell Magnetic Bead Panel (ref. HSTCMAG-28SK, Millipore Corporation, Burlington, MA, USA), following the manufacturer’s instructions. Samples were acquired in a Luminex platform (LABScan 100) using xPONENT v4.2 as acquisition and analysis software. According to the manufacturer’s instructions, kynurenine was quantified from plasma samples preserved at −80 °C using the Kynurenine ELISA pack (ref. ISE-2227, ImmuSmol, Bordeaux, France) and a Dynex DS2^®^ ELISA analyser (Dynex Technologies, Chantilly, VA, USA) as a colourimetric reader.

### 5.11. Statistical Analysis

Clinical and demographical characteristics of patients were summarized using mean and standard deviation in quantitative variables and total number and percentage for the description of qualitative variables. To assess the differences in the parameters between AG and TG in each period of time, the *t*-test was employed in those normalities distributed according to the Shapiro–Wilk test, selecting its version for equal or unequal variances depending on the result of the Levene test. In other cases, the Mann–Whitney U test was used. Moreover, a longitudinal study was performed to check the evolution of parameters in AG with respect to its T0 value. This analysis was carried out with linear mixed-effect (LME) models introducing time as a fixed effect and patient as a random effect. The *p*-values < 0.05 were regarded as statistically significant. Results were represented by box plots with Tukey whiskers and mean with standard error bars using Graphpad Prism (v. 6.01) [45]. R statistical software (v. 3.6.2) was used to perform all the analyses, and LME models were done with the nlme package [46].

## Figures and Tables

**Table 1:** Individual characteristics of the 20 allergic patients (AG) at the time of inclusion in the study (**Demographics, Diagnostic**) and concerning treatment (**Treatment**). The columns show: Male, M/Female, F (**Sex**); years (**Age**); occupational exposure to stings (**Employment**); any proven allergic sensitization to aeroallergens or foods: yes/no (**Atopy**); severity of anaphylaxis after spontaneous sting according to EAACI classification (**AX-Grade)**; risk of clonal mast cell activation (**REMA-score**); concentration of AmV extract in µg/mL at which the intradermal test was positive (**ID-test**); specific IgE value to Apis as IU/mL (**sIgE Apis**); specific IgG4 value to Apis as µg/mL (**sIgG4 Apis**); µg AmV for maintenance (**BVIT Dose)**; number and severity grade (n/n)of systemic reaction according to EAACI grading (**SR to BVIT**); and response (positive/negative/not done) to the sting challenge test (**SCT**).

	Allergic Patients											
	Demographics			Diagnostic						Treatment		
*n*	Sex	Age	Employment	Atopy	AX Grade	REMA Score	ID Test	sIgE Apis	sIgG4 Apis	BVIT Dose	SR to BVIT	SCT
1	F	52	Beekeeper	No	3	<2	0.01	36.5	0.29	200	-	ND
2	M	65	Builder	No	3	<2	0.001	11.7	4.48	100	-	Neg
3	M	49	Truck Driver	No	2	<2	0.01	6.96	0.09	100	-	Neg
4	M	43	Harvester	Yes	3	>2	0.01	0.29	0.02	100	-	Neg
5	M	28	Beekeeper	No	3	<2	0.001	6.75	0.16	200	-	Neg
6	F	25	Beekeeper	No	2	<2	0.001	3.48	0.07	200	1 (1)	Neg
7	M	46	Beekeeper	Yes	3	<2	Neg	7.98	0.18	300	-	Neg
8	F	68	Retired	Yes	3	<2	0.01	0.37	0.11	100	-	Neg
9	F	41	Beekeeper	No	2	<2	0.1	0.54	0.03	100	-	Neg
10	M	30	Beekeeper	No	3	>2	Neg	0.24	0.00	150	1 (3)	Neg
11	M	18	Beekeeper	No	3	<2	0.01	238	0.90	200	-	Neg
12	M	45	Beekeeper	No	2	<2	0.01	0.22	0.002	100	-	Neg
13	F	54	Teacher	Yes	2	<2	0.1	8.4	0.5	100	-	Neg
14	M	32	Harvester	Yes	3	<2	0.01	27.1	1.31	300	-	Neg
15	M	48	Beekeeper	Yes	3	<2	0.01	16	0.19	300	-	Neg
16	M	50	Beekeeper	Yes	2	<2	0.01	24.6	0.99	300	-	Neg
17	M	51	Beekeeper	No	3	<2	0.1	1.16	1.41	200	-	Neg
18	M	52	Beekeeper	No	3	<2	0.01	5.88	0.09	300	-	Neg
19	M	53	Sealer	Yes	2	<2	0.001	57.8	0.22	100	-	Neg
20	M	54	Harvester	Yes	3	<2	0.1	8.09	1.34	300	-	Neg

**Table 2:** Individual characteristics of the 20 tolerant beekeepers (TG) at the time of inclusion in the study (**Demographics, Diagnostic**). The columns show: Male, M/Female, F (**Sex**); years (**Age**); atopy (yes/no); stings per year; specific IgE value to Apis as IU/mL (**sIgE Apis**); specific IgG4 value to Apis as µg/mL (**sIgG4 Apis**).

	Tolerant Group					
	Demographics		Diagnostic			
*n*	Sex	Age	Atopy	Stings per Year	sIgE Apis	sIgG4 Apis
1	F	52	Yes	>200	0.21	21
2	M	65	No	>200	0.18	18.94
3	M	49	No	>200	0.94	21.98
4	M	43	No	50	7.86	5.37
5	M	28	No	>200	0.25	27.87
6	F	28	No	>200	1.33	13.44
7	M	46	No	>200	0.48	24.58
8	F	68	No	50	1.83	2.74
9	F	41	No	>200	0.28	17.13
10	M	30	No	>200	1.33	23.38
11	M	26	No	>200	1.27	29.43
12	M	45	No	>200	1.67	3.689
13	F	54	No	>200	0.61	27.30
14	M	32	Yes	>200	0.61	29.58
15	M	48	No	>200	0.24	21.38
16	M	50	Yes	50	0.64	15.00
17	M	51	No	50	4.47	0.66
18	M	42	No	>200	1.1	768
19	M	56	No	>200	0.73	12.3
20	M	57	No	>200	0.07	256

## Data Availability

The data presented in this study are available on request from the corresponding author. The data are not publicly available due to ethic reasons.

## Supplementary Materials

The following supporting information can be downloaded at: https://www.mdpi.com/article/10.3390/toxins14070426/s1. Figure S1: Dosing scheme for BVIT updosing; Figure S2: Basophil activation test gatting strategy. Figure S3: Th1/Th2/Th17 gatting strategy. Figure S4: Treg gatting strategy. Table S1: Mean value and standard deviation (in braquets) of every outcome for allergic patients (AG) at baseline (T0), after first cluster of BVIT (T1), after completing updosing (T2) and at the end of the first year of BVIT (T3); and for the tolerant beekeepers (TG) in a single time. T cell populations (both effector and regulatory) are expressed in %; basophil activation % after priming with two venom doses; interleukynes in pg/ml; kynurenine in ng/mL, sIgE in IU/mL and sIgG4 in µg/mL.

## References

[1] Sturm G.J., Varga E.M., Roberts G., Mosbech H., Bilò M.B., Akdis C.A., Antolín-Amérigo D., Cichocka-Jarosz E., Gawlik R., Jakob T. (2018). EAACI guidelines on allergen immunotherapy: Hymenoptera venom allergy. Allergy.

[2] Varga E.M., Kausar F., Aberer W., Zach M., Eber E., Durham S.R., Shamji M.H. (2013). Tolerant beekeepers display venom-specific functional IgG4 antibodies in the absence of specific IgE. J. Allergy Clin. Immunol..

[3] Ruiz-Leon B., Navas A., Serrano P., Espinazo M., Guler I., Alonso C., Jurado A., Moreno-Aguilar C. (2021). Helios negative Regulatory T-cells as a key factor of immune tolerance in non-allergic beekeepers. J. Investig. Allergol. Clin. Immunol..

[4] Kemeny D.M., Lessof M.H., Patel S., Youlten L.J., Williams A., Lambourn E. (1989). IgG and IgE antibodies after immunotherapy with bee and wasp venom. Int. Arch. Allergy Appl. Immunol..

[5] Ozdemir C., Kucuksezer U.C., Akdis M., Akdis C.A. (2011). Mechanisms of immunotherapy to wasp and bee venom. Clin. Exp. Allergy.

[6] Thornton A.M., Shevach E.M. (2019). Helios: Still behind the clouds. Immunology.

[7] Thornton A.M., Lu J., Korty P.E., Kim Y.C., Martens C., Sun P.D., Shevach E.M. (2019). Helios^+^ and Helios^−^ Treg subpopulations are phenotypically and functionally distinct and express dissimilar TCR repertoires. Eur. J. Immunol..

[8] Noval Rivas M., Chatila T.A. (2016). Regulatory T cells in allergic diseases. J. Allergy Clin. Immunol..

[9] Boonpiyathad T., Sözener Z.C., Akdis M., Akdis C.A. (2020). The role of Treg cell subsets in allergic disease. Asian. Pac. J. Allergy Immunol..

[10] Lan F., Zhang N., Bachert C., Zhang L. (2020). Stability of regulatory T cells in T helper 2-biased allergic airway diseases. Allergy.

[11] Syed A., Garcia M.A., Lyu S.C., Bucayu R., Kohli A., Ishida S., Berglund J.P., Tsai M., Maecker H., O’Riordan G. (2014). Peanut oral immunotherapy results in increased antigen-induced regulatory T-cell function and hypomethylation of forkhead box protein 3 (FOXP3). J. Allergy Clin. Immunol..

[12] Boonpiyathad T., Sokolowska M., Morita H., Rückert B., Kast J.I., Wawrzyniak M., Sangasapaviliya A., Pradubpongsa P., Fuengthong R., Thantiworasit P. (2019). Der p 1-specific regulatory T-cell response during house dust mite allergen immunotherapy. Allergy.

[13] Urra J.M., Cabrera C.M., Alfaya T., Feo-Brito F. (2016). Agreement of skin test with IL-4 production and CD40L expression by T cells upon immunotherapy of subjects with systemic reactions to Hymenoptera stings. Mol. Immunol..

[14] Riccio A.M., Saverino D., Pesce G., Rogkakou A., Severino M., Bonadonna P., Ridolo E., Mauro M., Canonica G.W., Bagnasco M. (2012). Effects of different up-dosing regimens for hymenoptera venom immunotherapy on serum CTLA-4 and IL-10. PLoS ONE.

[15] Urra J.M., Carrasco P., Feo-Brito F., De La Roca F., Guerra F., Cabrera C.M. (2014). Immunotherapy reduces CD40L expression and modifies cytokine production in the CD4 cells of pollen allergy patients. J. Investig. Allergol. Clin. Immunol..

[16] Moitra S., Datta A., Mondal S., Hazra I., Faruk S.M.O., Das P.K., Basu A.K., Tripathi S.K., Chaudhuri S. (2017). Modulation of regulatory T cells by intranasal allergen immunotherapy in an experimental rat model of airway allergy. Int. Immunopharmacol..

[17] Dioszeghy V., Mondoulet L., Puteaux E., Dhelft V., Ligouis M., Plaquet C., Dupont C., Benhamou P.H. (2017). Differences in phenotype, homing properties and suppressive activities of regulatory T cells induced by epicutaneous, oral or sublingual immunotherapy in mice sensitized to peanut. Cell Mol. Immunol..

[18] Anvari S., Watkin L., Rajapakshe K., Hassan O., Schuster K., Coarfa C., Davis C.M. (2021). Memory and naïve gamma delta regulatory T-cell gene expression in the first 24-weeks of peanut oral immunotherapy. Clin. Immunol..

[19] Wang S.H., Zissler U.M., Buettner M., Heine S., Heldner A., Kotz S., Pechtold L., Kau J., Plaschke M., Ullmann J.T. (2021). An exhausted phenotype of T_H_ 2 cells is primed by allergen exposure, but not reinforced by allergen-specific immunotherapy. Allergy.

[20] Kucuksezer U.C., Ozdemir C., Cevhertas L., Ogulur I., Akdis M., Akdis C.A. (2020). Mechanisms of allergen-specific immunotherapy and allergen tolerance. Allergol. Int..

[21] Zhao L., Zhou X., Zhou X., Wang H., Gu L., Ke Y., Zhang M., Ji X., Yang X. (2020). Low expressions of PD-L1 and CTLA-4 by induced CD4^+^CD25^+^ Foxp3^+^ Tregs in patients with SLE and their correlation with the disease activity. Cytokine.

[22] Bussmann C., Xia J., Allam J.P., Maintz L., Bieber T., Novak N. (2010). Early markers for protective mechanisms during rush venom immunotherapy. Allergy.

[23] Francis J.N., James L.K., Paraskevopoulos G., Wong C., Calderon M.A., Durham S.R., Till S.J. (2008). Grass pollen immunotherapy: IL-10 induction and suppression of late responses precedes IgG4 inhibitory antibody activity. J. Allergy Clin. Immunol..

[24] Boonpiyathad T., Satitsuksanoa P., Akdis M., Akdis C.A. (2019). Il-10 producing T and B cells in allergy. Semin. Immunol..

[25] Golebski K., Layhadi J.A., Sahiner U., Steveling-Klein E.H., Lenormand M.M., Li R.C.Y., Bal S.M., Heesters B.A., Vilà-Nadal G., Hunewald O. (2021). Induction of IL-10-producing type 2 innate lymphoid cells by allergen immunotherapy is associated with clinical response. Immunity.

[26] Pallotta M.T., Rossini S., Suvieri C., Coletti A., Orabona C., Macchiarulo A., Volpi C., Grohmann U. (2021). Indoleamine 2,3-dioxygenase 1 (IDO1): An up-to-date overview of an eclectic immunoregulatory enzyme. FEBS J..

[27] Huang Y.S., Ogbechi J., Clanchy F.I., Williams R.O., Stone T.W. (2020). IDO and Kynurenine Metabolites in Peripheral and CNS Disorders. Front. Immunol..

[28] Tanaka M., Tóth F., Polyák H., Szabó Á., Mándi Y., Vécsei L. (2021). Immune Influencers in Action: Metabolites and Enzymes of the Tryptophan-Kynurenine Metabolic Pathway. Biomedicines.

[29] Mamessier E., Birnbaum J., Dupuy P., Vervloet D., Magnan A. (2006). Ultra-rush venom immunotherapy induces differential T cell activation and regulatory patterns according to the severity of allergy. Clin. Exp. Allergy.

[30] Schuerwegh A.J., De Clerck L.S., Bridts C.H., Stevens W.J. (2001). Wasp venom immunotherapy induces a shift from IL-4-producing towards interferon-gamma-producing CD4+ and CD8+ T lymphocytes. Clin. Exp. Allergy.

[31] Chaker A.M., Shamji M.H., Dumitru F.A., Calderon M.A., Scadding G.W., Makatsori M., Jones I., He Q.A., Subramanian K.K., Arm J.P. (2016). Short-term subcutaneous grass pollen immunotherapy under the umbrella of anti-IL-4: A randomized controlled trial. J. Allergy Clin. Immunol..

[32] Zielen S., Gabrielpillai J., Herrmann E., Schulze J., Schubert R., Rosewich M. (2018). Long-term effect of monophosphoryl lipid A adjuvanted specific immunotherapy in patients with grass pollen allergy. Immunotherapy.

[33] Tuzlak S., Dejean A.S., Iannacone M., Quintana F.J., Waisman A., Ginhoux F., Korn T., Becher B. (2021). Repositioning T_H_ cell polarization from single cytokines to complex help. Nat. Immunol..

[34] Sahiner U.M., Durham S.R. (2019). Hymenoptera Venom Allergy: How Does Venom Immunotherapy Prevent Anaphylaxis from Bee and Wasp Stings?. Front. Immunol..

[35] Demšar Luzar A., Korošec P., Košnik M., Zidarn M., Rijavec M. (2021). *Hymenoptera* Venom Immunotherapy: Immune Mechanisms of Induced Protection and Tolerance. Cells.

[36] Abbas A.K., Lichtman A.H., Pillai S., Jeremy B. (2022). B Cell Activation and Antibody Production in Cellular and Molecular Immunology.

[37] Rodríguez Trabado A., Cámara Hijón C., Ramos Cantariño A., Romero-Chala S., García-Trujillo J.A., Fernández Pereira L.M. (2016). Short-, Intermediate-, and Long-Term Changes in Basophil Reactivity Induced by Venom Immunotherapy. Allergy Asthma Immunol. Res..

[38] Peternelj A., Silar M., Erzen R., Kosnik M., Korosec P. (2008). Basophil sensitivity in patients not responding to venom immunotherapy. Int. Arch. Allergy Immunol..

[39] Eržen R., Košnik M., Silar M., Korošec P. (2012). Basophil response and the induction of a tolerance in venom immunotherapy: A long-term sting challenge study. Allergy.

[40] Behrends J., Schwager C., Hein M., Scholzen T., Kull S., Jappe U. (2021). Innovative robust basophil activation test using a novel gating strategy reliably diagnosing allergy with full automation. Allergy.

[41] Alvarez-Twose I., González de Olano D., Sánchez-Muñoz L., Matito A., Esteban-López M.I., Vega A., Mateo M.B., Alonso Díaz de Durana M.D., de la Hoz B., Del Pozo Gil M.D. (2010). Clinical, biological, and molecular characteristics of clonal mast cell disorders presenting with systemic mast cell activation symptoms. J. Allergy Clin. Immunol..

[42] Muraro A., Fernandez-Rivas M., Beyer K., Cardona V., Clark A., Eller E., Hourihane J.O.B., Jutel M., Sheikh A., Agache I. (2018). The urgent need for a harmonized severity scoring system for acute allergic reactions. Allergy.

[43] Moreno C., Barasona M.J., Serrano P., Justicia J.L., Ruz J.M., Guerra F. (2011). Alternating Polistes-Vespula venom immunotherapy: A therapeutic strategy to resolve a diagnostic deficiency. J. Investig. Allergol. Clin. Immunol..

[44] Axen R., Dreven H. (1988). A new laboratory diagnostic system applied to allergy testing. Allergy Proc..

[45] R Core Team (2019). R: A Language and Environment for Statistical Computing.

[46] Pinheiro J., Bates D., DebRoy S., Sarkar D., R Core Team (2019). nlme: Linear and Nonlinear Mixed Effects Models.

