# Supplementary material for: Natural and Induced Tolerance to Hymenoptera Venom: A Single Mechanism?
Source: Toxins (Basel). 2022 Jun 22;14(7):426. doi: 10.3390/toxins14070426 (PMC9320229; doi:10.3390/toxins14070426)
Supplement: Supplementary file 1 [file toxins-14-00426-s001.zip › toxins-1764590-supplementary.pdf]

toxins

Supplementary material

Natural and Induced Tolerance to Hymenoptera Venom: A Single Mechanism?

Ana Navas, Berta Ruiz-Leon, Pilar Serrano, Manuel Martí, M Luisa Espinazo, Nadine Blanco, Juan Molina, Corona Alonso, Aurora Jurado and Carmen Moreno-Aguilar

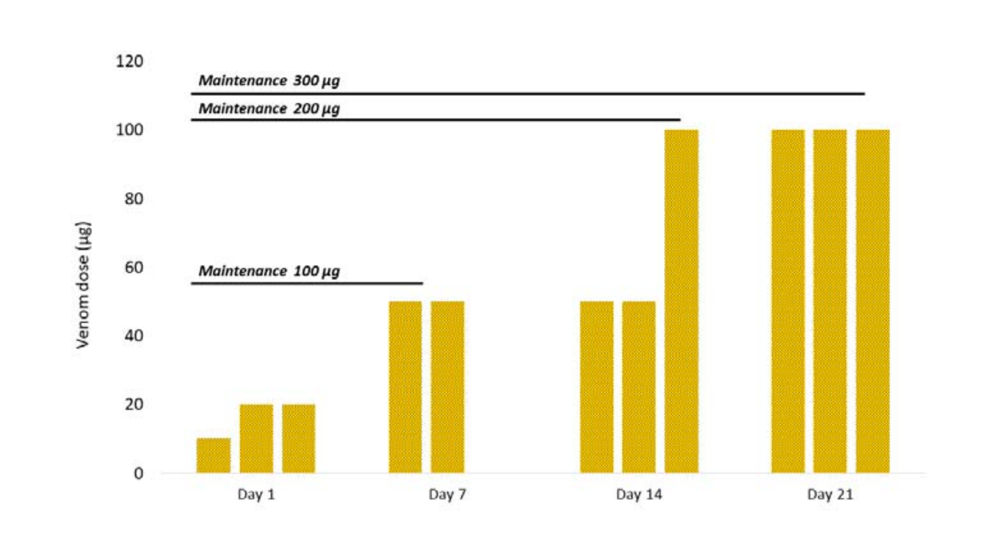

Figure S1. Dosing scheme for BVIT updosing. Each column represents an individual dose, separated 30 minutes from the next dose of the same cluster. The AG subjects receiving 100 µg maintenance did the updosing in two visits, the 200 µg subjects in three and the 300 µg subjects in four. At the end of each cluster, patients remained under observation for two hours.

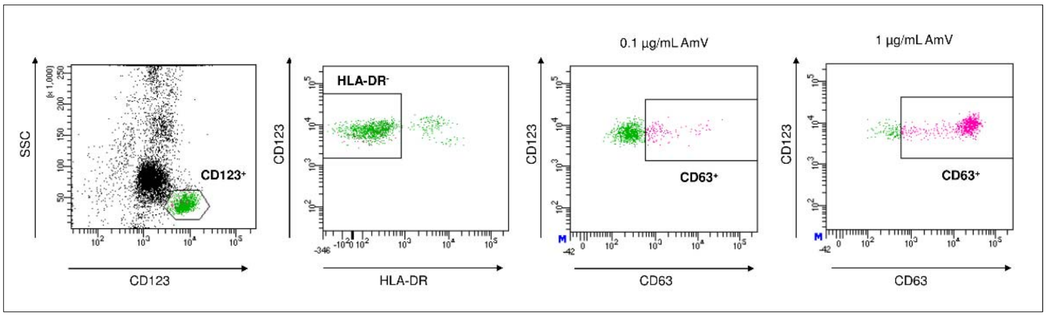

Figure S2. Basophil activation test gating strategy. SSC, Side scatter. The identification of basophils was performed according to the expression of CD123 and HLA-DR markers (CD123+HLA-DR-), while those degranulated (pink) were identified according to the expression of CD63 marker (CD63+). Results from total peripheral blood cells stimulated with 0.1 and 1 µg/mL of *Apis mellifera* venom (AmV) are displayed.

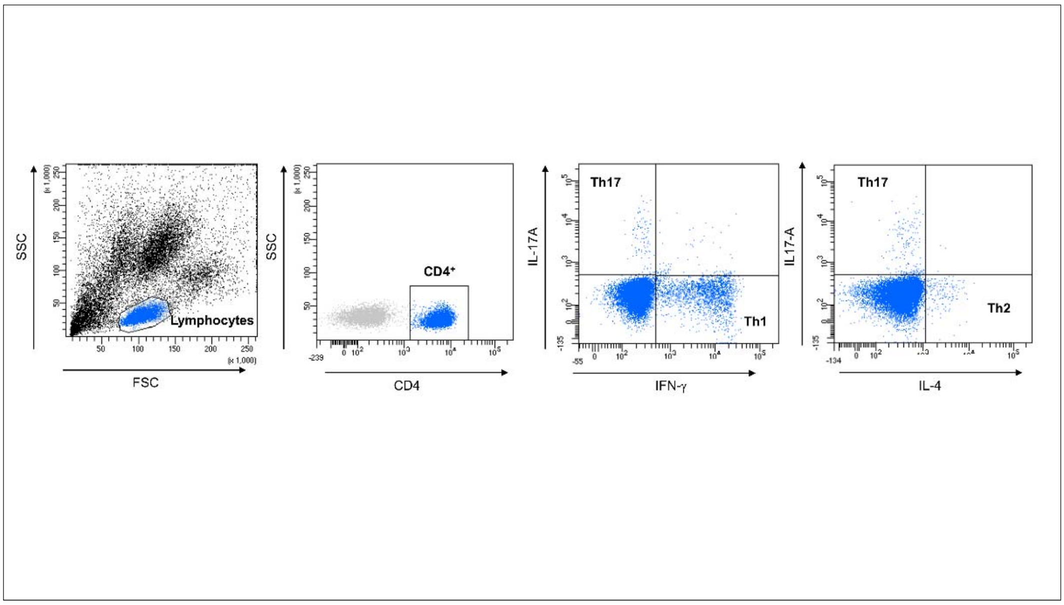

Figure S3. Th1/Th2/Th17 gatting strategy. FSC, Forward scatter; SSC; Side scatter. The identification of Th1/Th2/Th17 subsets was performed according to the expression of CD4 (blue) and the production of IFN-γ, IL-4 and IL-17 cytokines. Results from total peripheral blood cells stimulated with phorbol 12-myristate 13-acetate at 50 ng/mL and ionomycin calcium salt at 1 μg/mL are displayed.

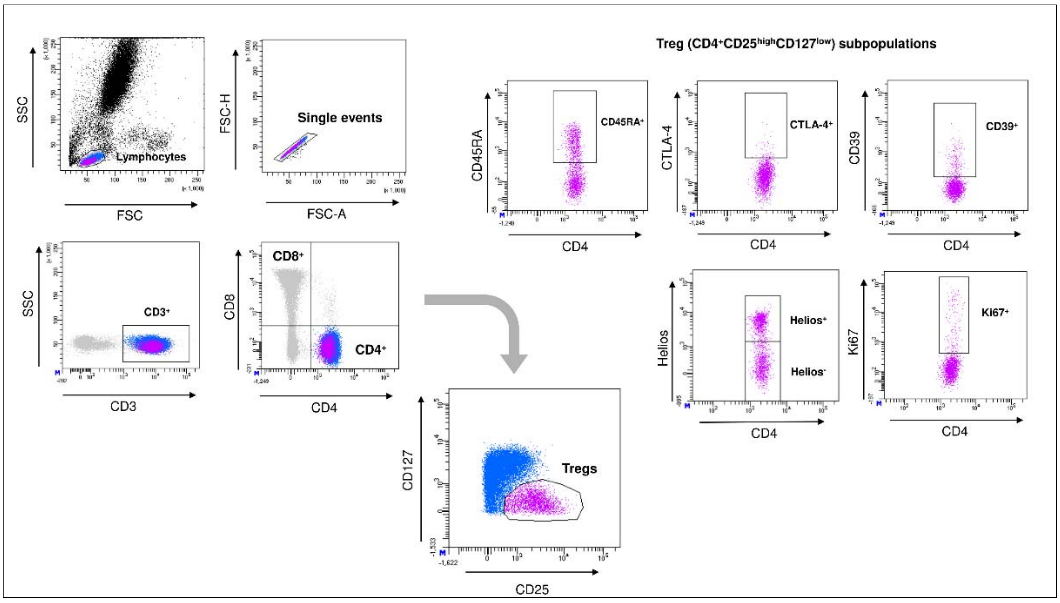

Figure S4. Treg gatting strategy. FSC, forward scatter; SSC, Side scatter. The phenotype of Treg ($CD4^{+}CD25^{high}CD127^{low}$) subpopulations from total peripheral blood was performed according the gating strategy here displayed.

Table S1. Mean value and standard deviation (in braquets) of every outcome for allergic patients (**AG**) at baseline (**T0**), after first cluster of BVIT (**T1**), after completing updosing (**T2**) and at the end of the first year of BVIT (**T3**); and for the tolerant beekeepers (**TG**) in a single time. T cell populations (both effector and regulatory) are expressed in %; basophil activation % after priming with two venom doses; interleukynes in pg/ml; kynurenine in ng/ml, sIgE in IU/ml and sIgG4 in μg/ml

		AG				TG
		**T0**	**T1**	**T2**	**T3**	
**T effector cells**	**CD4⁺ Th1**	12.08 (8.11)	10.82 (6.83)	12.55 (7.51)	14.59 (5.41)	6.85 (3.96)
	**CD4⁺ Th2**	0.82 (0.78)	0.93 (0.94)	0.90 (0.85)	1.39 (0.75)	0.34 (0.66)
**T regulatory cells**	**$CD4^+/CD25^{high}/CD127^{low}$**	8.56 (1.61)	8.73 (1.77)	8.43 (1.66)	7.35 (2.08)	8.28 (1.75)
	**Treg CTLA-4^+**	12.25 (10,68)	10.13 (7.31)	12.25 (7.74)	4.51 (4.95)	26.82 (11.85)
	**Treg $Helios^-$**	69.90 (19.04)	73.58 (15.50)	69.38 (18.11)	81.81 (19.32)	86.48 (11.47)
**Basophil Activation %**	**0.1 μg**	5.27 (10.35)	7.21 (16.42)	6.72 (11.75)	2.64 (3.26)	0.66 (0.67)
	**1 μg**	54.71 (27.83)	49.80 (29.07)	50.79 (27.18)	22.36 (21,15)	15,62 (20,77)
**Interleukynes**	**IL-4**	393.35 (405.43)	515.80 (449.78)	732.08 (762.62)	1,634.38 (945.46)	266.98 (166.63)
	**IL-10**	21.96 (15.17)	25.32 (25.23)	53.82 (47.92)	26.11 (21.36)	56.20 (31.43)
	**Kynurenine**	0.61 (0.21)	0.54 (0.21)	4.78 (3.19)	6.98 (2.42)	0.70 (0.79)
**Immunoglobulin E**	**sIgE AmV**	23.10 (52.67)	31.74 (71.55)	37.52 (73.41)	11.09 (14.48)	1.32 (1.85)
	**sIgE rApi m 1**	10.19 (23.22)	13.62 (31.20)	23.16 (55.98)	3.61 (7.12)	0.27 (0.29)
	**sIgE rApi m 2**	0.57 (1.52)	0.53 (1.35)	7.81 (20.83)	0.95 (2.11)	0.42 (0.41)
	**sIgE rApi m 3**	1.05 (2.71)	1.00 (2.50)	6.51 (13.07)	0.51 (0.89)	0.04 (0.16)
	**sIgE Api m 4**	0.85 (1.63)	1.25 (2.07)	1.89 (2.70)	0.33 (0,58)	0.07 (0,11)
	**sIgE rApi m 5**	2.34 (4.73)	2.21 (4.27)	4.81 (6.90)	1.36 (2.00)	0.09 (0.10)
	**sIgE rApi m 10**	11.81 (28.85)	12.52 (27.80)	20.21 (44.56)	9.03 (19.74)	0.31 (0.47)
**Immunoglobulin G4**	**sIgG4 AmV**	0.62 (1.03)	0.80 (1.78)	5.68 (6.63)	5.57 (3.95)	66.99 (173.61)
	**sIgG4 rApi m 1**	0.47 (1.01)	0.44 (0.89)	4.12 (6.37)	3.44 (2.50)	45.13 (125.15)
	**sIgG4 rApi m 2**	0.01 (0.04)	0.02 (0.05)	0.60 (2.42)	0.55 (0.81)	6.71 (5.51)
	**sIgG4 rApi m 3**	0.04 (0.15)	0.03 (0.10)	0.21 (0.68)	0.07 (0.15)	1.22 (1.80)
	**sIgG4 Api m 4**	0.09 (0.17)	0.19 (0.45)	1.71 (2.79)	0.24 (0.44)	7.82 (7.37)
	**sIgG4 rApi m 5**	0.02 (0.02)	0.02 (0.03)	0.08 (0.12)	0.06 (0.09)	1.37 (1.73)
	**sIgG4 rApi m 10**	0.02 (0.03)	0.02 (0.03)	0.16 (0.29)	0.04 (0.07)	1.56 (2.80)
